# Screw tightness and stripping rates vary between biomechanical researchers and practicing orthopaedic surgeons

**DOI:** 10.1186/s13018-021-02800-z

**Published:** 2021-10-26

**Authors:** James W. A. Fletcher, Verena Neumann, Lisa Wenzel, Boyko Gueorguiev, R. Geoff Richards, Harinderjit S. Gill, Michael R. Whitehouse, Ezio Preatoni

**Affiliations:** 1 Applied Biomechanics Suite, Department for Health, University of Bath, Claverton Down, Bath, BA2 7AY UK; 2 AO Research Institute Davos, Davos, Switzerland; 3 Department of Trauma Surgery, Trauma Center Murnau, Professor-Küntscher-Str. 8, 82418 Murnau, Germany; 4 Department of Mechanical Engineering, University of Bath, Bath, UK; 5 Centre for Therapeutic Innovation, University of Bath, Bath, UK; 6 Musculoskeletal Research Unit, Translational Health Sciences, Bristol Medical School, Southmead Hospital, 1st Floor Learning & Research Building, Bristol, UK; 7 National Institute for Health Research Bristol Biomedical Research Centre, University Hospitals Bristol NHS Foundation Trust and University of Bristol, Bristol, UK

**Keywords:** Researcher, Screw, Stripping rate, Surgeon, Tightness, Torque

## Abstract

**Background:**

Screws are the most frequently inserted orthopaedic implants. Biomechanical, laboratory-based studies are used to provide a controlled environment to investigate revolutionary and evolutionary improvements in orthopaedic techniques. Predominantly, biomechanical trained, non-surgically practicing researchers perform these studies, whilst it will be orthopaedic surgeons who will put these procedures into practice on patients. Limited data exist on the comparative performance of surgically and non-surgically trained biomechanical researchers when inserting screws. Furthermore, any variation in performance by surgeons and/or biomechanical researchers may create an underappreciated confounder to biomechanical research findings. This study aimed to identify the differences between surgically and non-surgically trained biomechanical researchers’ achieved screw tightness and stripping rates with different fixation methods.

**Methods:**

Ten orthopaedic surgeons and 10 researchers inserted 60 cortical screws each into artificial bone, for three different screw diameters (2.7, 3.5 and 4.5 mm), with 50% of screws inserted through plates and 50% through washers. Screw tightness, screw hole stripping rates and confidence in screw purchase were recorded. Three members of each group also inserted 30 screws using an augmented screwdriver, which indicated when optimum tightness was achieved.

**Results:**

Unstripped screw tightness for orthopaedic surgeons and researchers was 82% (*n* = 928, 95% CI 81–83) and 76% (*n* = 1470, 95% CI 75–76) respectively (*p* < 0.001); surgeons stripped 48% (872/1800) of inserted screws and researchers 18% (330/1800). Using washers was associated with increased tightness [80% (95% CI 80–81), *n* = 1196] compared to screws inserted through plates [76% (95% CI 75–77), *n* = 1204] (*p* < 0.001). Researchers were more accurate in their overall assessment of good screw insertion (86% vs. 62%). No learning effect occurred when comparing screw tightness for the first 10 insertions against the last 10 insertions for any condition (*p* = 0.058–0.821). Augmented screwdrivers, indicating optimum tightness, reduced stripping rates from 34 to 21% (*p* < 0.001). Experience was not associated with improved performance in screw tightness or stripping rates for either group (*p* = 0.385–0.965).

**Conclusions:**

Surgeons and researchers showed different screw tightness under the same in vitro conditions, with greater rates of screw hole stripping by surgeons. This may have important implications for the reproducibility and transferability of research findings from different settings depending on who undertakes the experiments.

## Background

Screws are the most commonly used orthopaedic implant and are needed in the majority of orthopaedic operations. In current practice, non-locking screws require the user’s subjective assessment of the torque that should be applied to achieve optimum fixation. Analysis of surgical techniques has shown a concerning spectrum of abilities in creating adequate constructs for osteosynthesis [1]. Screw insertion is potentially deemed a trivial procedure; for example, in orthopaedic surgical training there are no specific quantitative assessments of screw insertion abilities [2]. With one exception [3], previous studies into insertion techniques and their effects have usually been limited by involving only one surgeon inserting all screws [4–8], or several surgeons each inserting only a few screws [9–17]. Only one study is in vivo, showing 36% of 225 inserted screws were stripped and required augmentation to salvage [17]. There are no existing studies comparing and contrasting the outcomes of non-surgical biomechanical researchers with those from surgeons, despite the numerous studies into screw fixation performed by the former [1]. Furthermore, limited data exist on the screw tightness commonly achieved by surgeons and researchers, and the effect on tightness from variations in parameters such as screw diameter [1, 3]. Given that biomechanical research is often performed by non-surgical researchers, differences in the abilities between surgical and non-surgical researchers could have considerable repercussions for the clinical transferability of findings generated by the latter.

This study was designed to assess the following comparisons between a sample of orthopaedic surgeons and biomechanical researchers, with the null hypothesis of there being no difference in their performance under any defined conditions. Comparisons were made to investigate any differences in the groups’ screw tightness and screw hole stripping rates when inserting screws into plates or through washers and when inserting different diameter screws. Additional comparisons were made to ascertain any difference between surgeons and researchers in: the reported confidence in screw insertions that had or had not stripped screw holes, the detection of stripping of screw holes, the presence of a learning effect when inserting screws, the impact of awareness of applied torque and whether indication of optimum tightness affected screw tightness and stripping rates.

## Methods

**Fig. 1:**
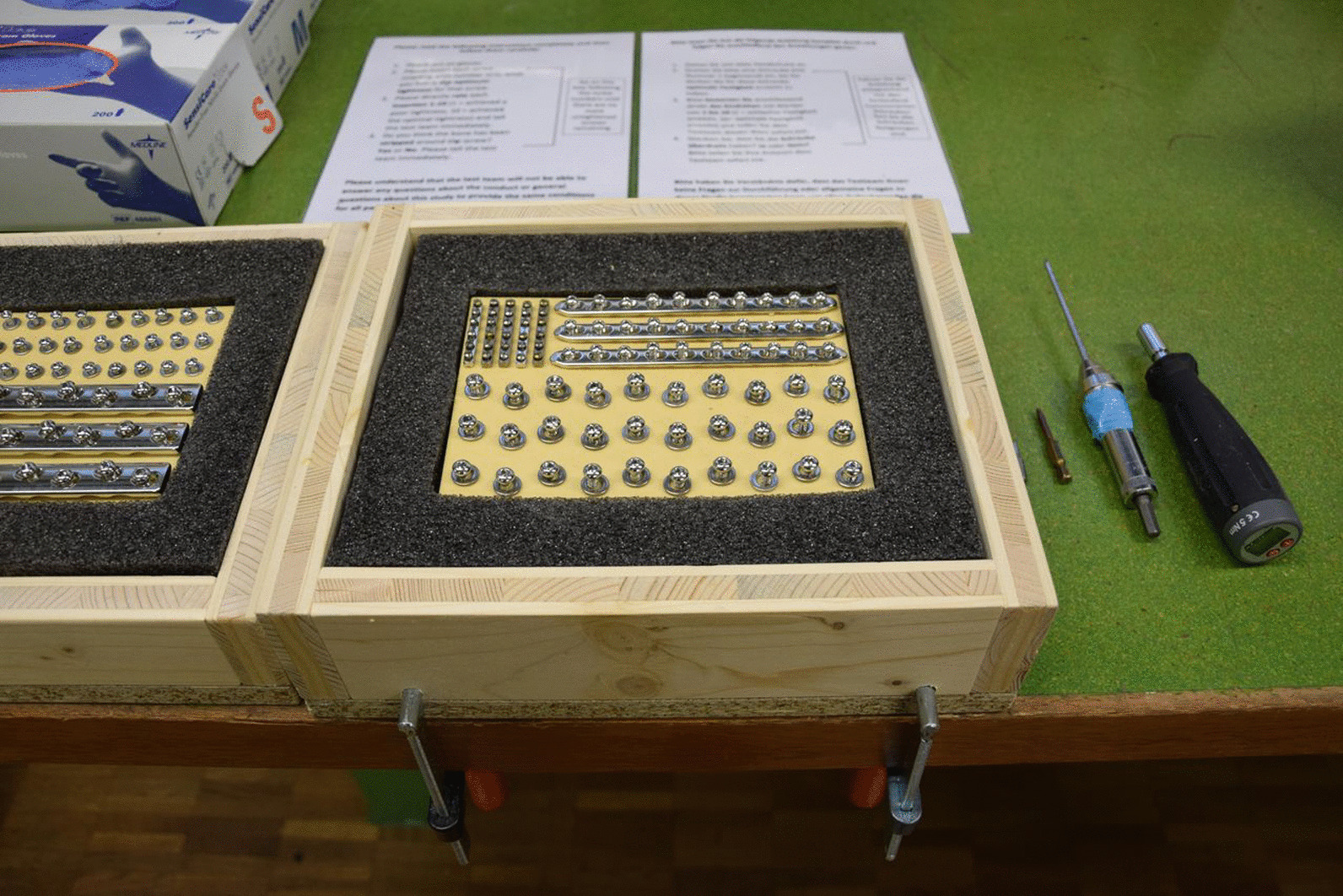
Apparatus set up for insertion testing, with foam base mimicking human tissue stiffness

Custom made testing apparatus was created for standardised screw insertion. Artificial bone sheets (Synbone, Zizers, Switzerland) were manufactured, 4 mm thick, with a density of 20 pounds per cubic foot (PCF). Using a milling machine (FP1, Deckel Maho GmbH, Pfronten, Germany), 90 drill holes were made perpendicularly in each of 40 sheets; each sheet contained 30 drill holes of 2.0 mm, 2.5 mm and 3.2 mm to receive 2.7 mm, 3.5 mm and 4.5 mm cortical screws, respectively. A wooden jig was created, containing a foam base to mimic the stiffness of human soft tissue (Fig. 1). Screw holes were made in the foam using the template so that screw threads would only engage in the artificial bone, whilst the remaining foam provided stiffness to the construct. Pilot testing had shown that 24 screws would be needed to detect a difference of 10 ± 12% in tightness with 80% power at a significance of 0.05; this was increased to 30 screws in case of experimental issues. All screws (De Puy Synthes, Zuchwil, Switzerland) were stainless steel, self-tapping and fully threaded. Participants were asked to insert a total of 180 screws, with 60 inserted for each of the three screw diameters: 30 through washers and 30 through plate holes of the respective size for that screw. To ensure that toggle from initial insertion was not introduced by participants and that all screw insertions were started in a similar fashion, two study investigators pre-inserted all screws 3 to 5 mm from the surface of the plate or washer before being tightened by the participant.

Ten visiting surgeons and 10 biomechanical researchers were recruited from the AO Research Institute Davos, Davos, Switzerland; participants gave informed consent for assessment of their techniques. The number of years of experience in their respective fields was recorded. All tests occurred with only the test participant and investigators present, to remove any confounding due to peer distractions [13]. Participants were blinded to the torque being applied. The ordering for the six testing conditions was randomised between participants using a simple sequence randomisation. Participants were given the same written instructions, including to wear unsterile, single layer nitrile gloves and to tighten each screw to what they determined to be the optimum tightness [3]; no specific technique was taught beforehand. Each screw was tightened using a torque measuring screwdriver (Premier STS103, Jack Sealey LTD., Bury St. Edmunds, UK), with the screwdriver bit changed to match the screw drive. Each screw was used 12 times, with screws and screwdriver bits changed if any macroscopic damage occurred. Participants were asked after every screw insertion whether they felt the screw hole had been stripped, and to rate their confidence in the screw’s holding ability from 1 to 10 (1 being very poor and 10 being optimal). After each screw was tightened, the stopping torque was recorded by a study investigator, with the participant blinded to the value. After all screws had been tightened by participants to the perceived optimum, investigators overtightened each screw to determine the stripping torque for that screw hole—defined as the maximum torque recordable for that screw in that screw hole. This was compared to the stopping torque to determine the screw tightness—as a ratio of stopping to stripping torque. If the stopping torque of the participant was greater than the stripping torque created by the investigator, it was recorded that the screw hole had been stripped by the participant; this enabled calculation of the stripping rate.

Following initial analysis, the participants with the 1st, 5th and 10th highest stripping rates from both the surgeons and researchers were asked to re-attend on a different day to insert a further 60 3.5 mm screws through plates. With these insertions, half were performed as per their normal technique, followed by half with participants unblinded to the applied torque, with the screwdriver (Premier STS103, Jack Sealey LTD., Bury St. Edmunds, UK) set to vibrate and alarm when the optimum tightness was reached; optimum tightness was set at 70% of the mean average stripping torque [3, 9, 10], which was calculated by mean averaging the stripping torque for all previous insertions of 3.5 mm screws.

### Statistical analysis

Statistical analysis was performed using unpaired, two-tailed *t* tests for comparisons of years of experience, screw tightness and stripping rates for surgeons and researchers, and paired two-tailed *t* tests for comparisons between tested variables: for plates and washers, for different screw diameters, for reported confidence for stripped and unstripped insertions, for the first ten screw insertions against the last ten screw insertions and for unaugmented and augmented screw insertions. Rates of screw hole stripping were compared using McNemar and Chi Squared tests. Bonferroni corrections were performed for cases of multiple comparisons, with adjusted values reported. Using the confidence values reported, the sensitivity, specificity and accuracy for screw hole stripping were calculated. The maximum and mean average Youden Indices were calculated. Results were considered significant at a level of significance of 0.05, and confidence intervals were calculated at 95%. Statistical tests were performed with IBM SPSS Statistics for Windows, version 20 (IBM SPSS Corp., Armonk, N.Y., USA). All data are available in an online repository [18].

## Results

A total of 3960 screw insertions were performed, with all available for analysis. Average experience was four years (range 1–19) for surgeons and 10 years (range 3–26) for researchers (*p* = 0.09).

**Fig. 2:**
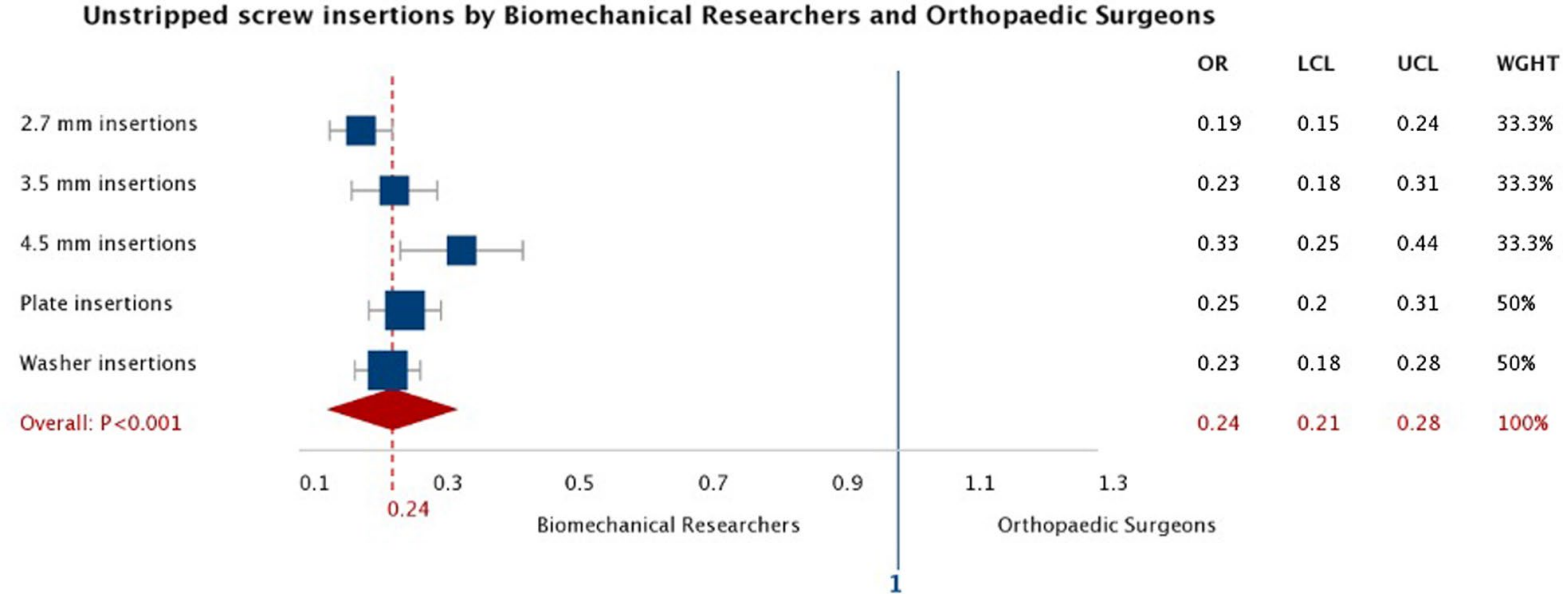
Forest plot of the Odds ratios for surgeons and researchers for unstripped screw insertion (*OR* odds ratio, *LCL* lower confidence level, *UCL* upper confidence level, *WGHT* weighting)

**Fig. 3:**
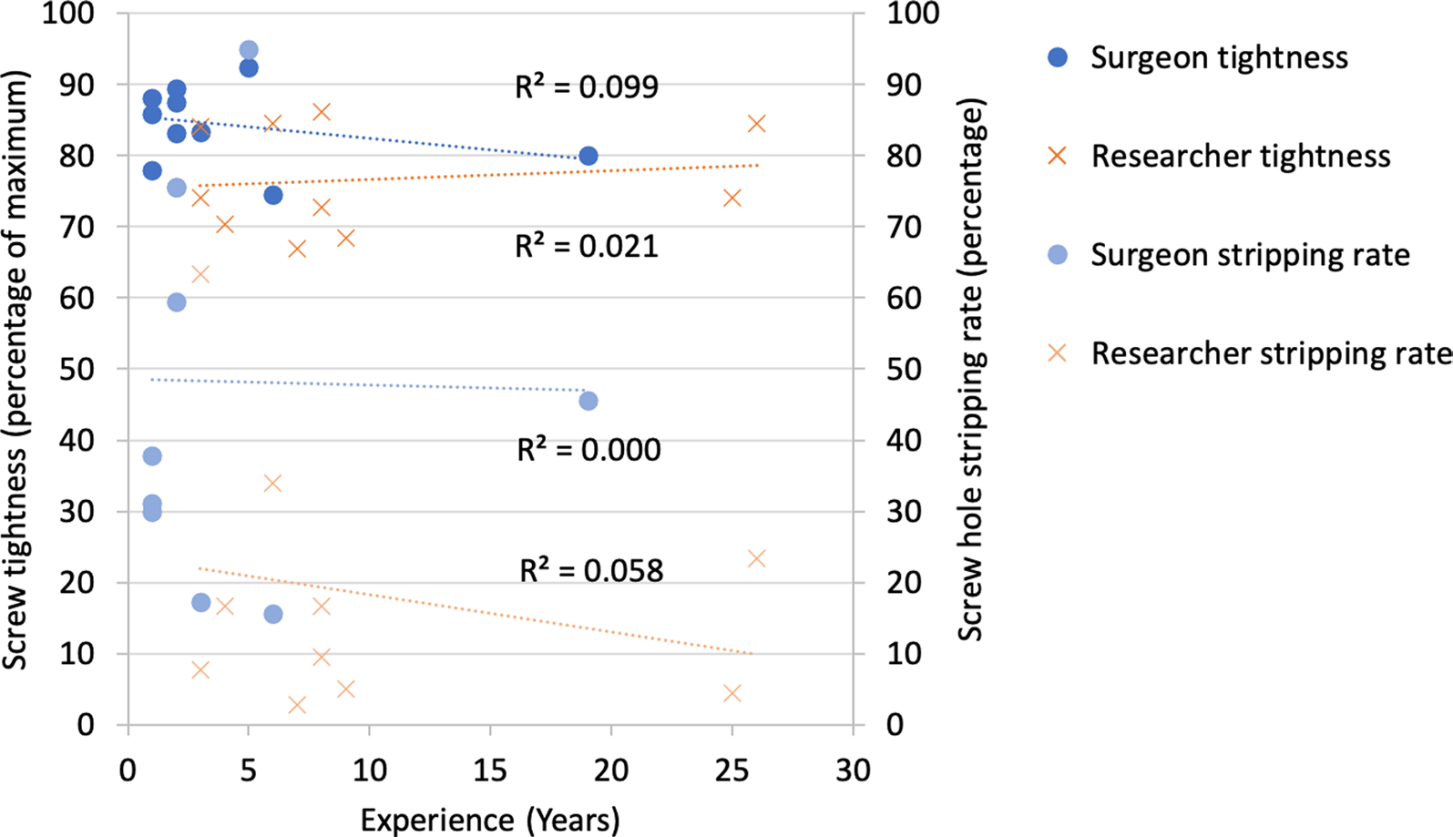
Screw tightness and stripping rates for each participant (10 surgeons and 10 researchers) compared with years of experience, with no significant associations seen

For all unstripped insertions, screw tightness was higher for surgeons [82% (95% CI 81–83), *n* = 928] than for researchers [76% (95% CI 75–76), *n* = 1470] (*p* < 0.001), with a greater stripping rate: 48% (872/1800) versus 18% (330/1800) (*p* < 0.001). Tightness and stripping rates for different screw diameters and plate and washer insertions are summarised in Table 1. Odds ratios for stripping under different conditions are shown in Fig. 2. Higher screw tightness was seen for screws inserted through washers compared to plates (*p* < 0.001). Lower tightness was seen with 4.5 mm insertions compared to 3.5 mm insertions for both surgeons (*p* < 0.001) and researchers (*p* = 0.04) and compared to 2.7 mm insertions for researchers (*p* < 0.001). Analysed separately, for surgeons and researchers, there was no association between experience and either screw tightness (*R*^2^ = 0.099, *p* = 0.385 and *R*^2^ = 0.021, *p* = 0.687) or stripping rates (*R*^2^ = 0.000, *p* = 0.965 and *R*^2^ = 0.058, *p* = 0.502) (Fig. 3).

**Table 1:** Tightness and stripping rates for researchers and surgeons under different testing conditions

	Number of insertions attempted	Number of unstripped insertions	Stripping rate (%)	Statistical difference in stripping rate	Unstripped screw tightness (%) (95% CI)	Statistical difference in tightness
All insertions						
All participants	3600	2400	33		78 (78–79)	
Surgeons	1800	928	48	*p* < 0.001	82 (81–83)	*p* < 0.001
Researchers	1800	1470	18		76 (75–76)	
Plate insertions						
All participants	1800	1204	33		76 (75–77)	
Surgeons	900	472	48	*p* < 0.001	82 (80–83)	*p* < 0.001
Researchers	900	732	19		72 (71–74)	
Washer insertions						
All participants	1800	1196	34		81 (80–81)	
Surgeons	900	458	49	*p* < 0.001	83 (82–84)	*p* < 0.001
Researchers	900	738	18		79 (78–80)	
2.7 mm insertions						
All participants	1200	670	44		79 (78–80)	
Surgeons	600	218	64	*p* < 0.001	83 (81–85)	*p* < 0.001
Researchers	600	452	25		77 (76–79)	
3.5 mm insertions						
All participants	1200	835	30		80 (79–81)	
Surgeons	600	331	45	*p* < 0.001	84 (83–85)	*p* < 0.001
Researchers	600	504	16		77 (76–78)	
4.5 mm insertions						
All participants	1200	885	26		77 (75–78)	
Surgeons	600	381	37	*p* < 0.001	80 (79–82)	*p* < 0.001
Researchers	600	504	16		74 (72–76)

**Fig. 4:**
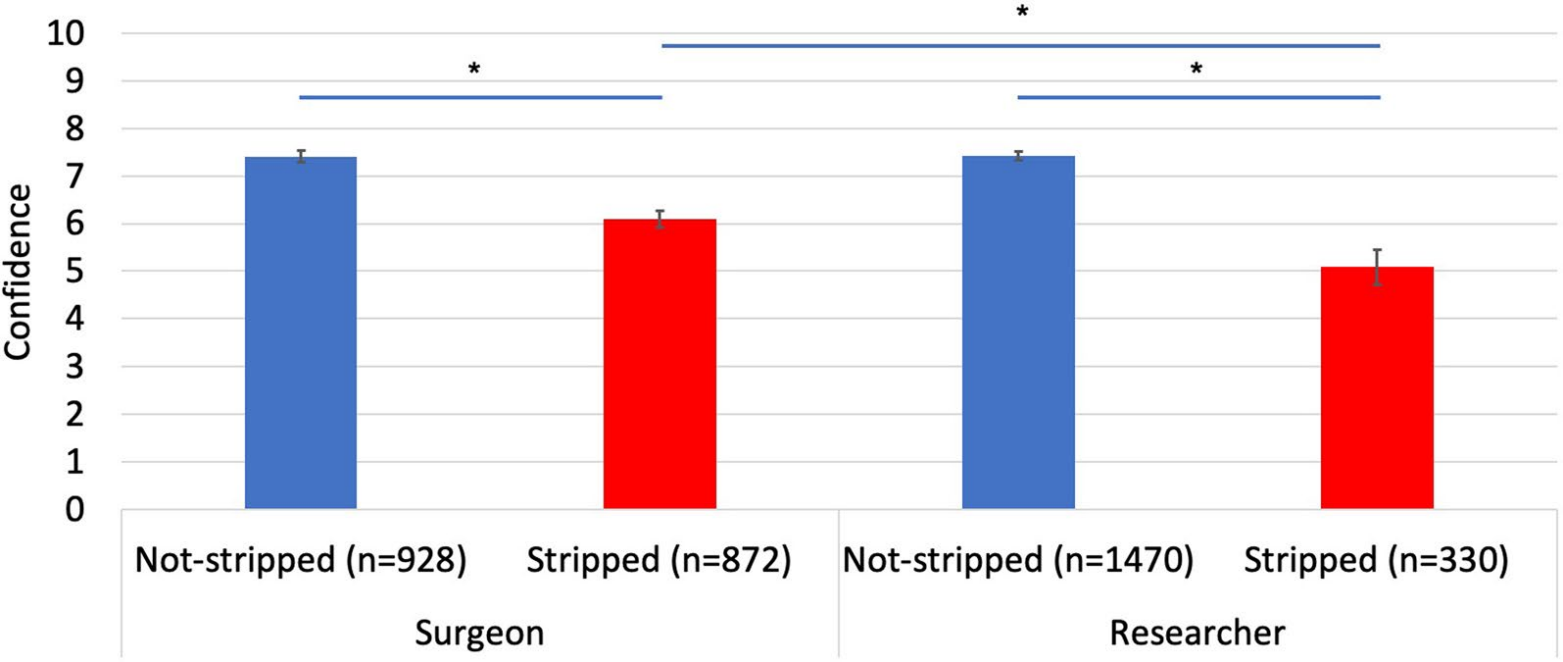
Confidence reported for unstripped and stripped insertions by surgeons and researchers (1 being very poor and 10 being optimal). Significant differences (*p* < 0.001) indicated with asterisk

**Fig. 5:**
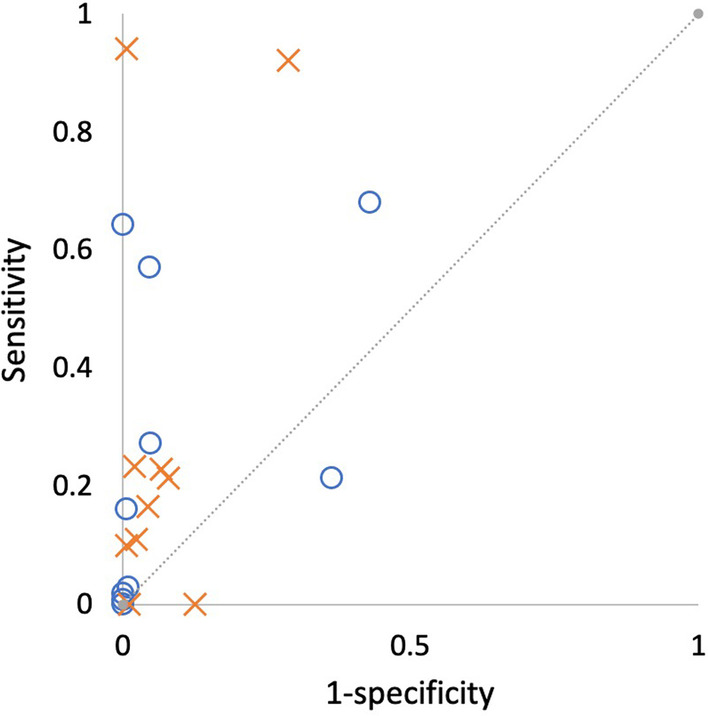
Receiver operating characteristic (ROC) curve for the diagnostic ability of surgeons and researchers for screw stripping. Surgeons indicated by blue circles and researchers by orange crosses

Both groups showed greater confidence in screw purchase for unstripped insertions compared to stripped insertions: surgeons—7.4 versus 6.1 (*p* < 0.001), researchers—7.4 versus 5.1 (*p* < 0.001) (Fig. 4). Researchers on average demonstrated a greater ability to correctly predict if a screw hole had been stripped compared to surgeons—sensitivity of 47% compared to 30% for surgeons (*p* < 0.001). Both groups were similarly specific when correctly predicting an unstripped screw hole—95% for researchers and 91% for surgeons (Fig. 5). The maximum and mean average Youden Indices for researchers were 0.94 and 0.22 and for surgeons were 0.64 and 0.17. Researchers also performed better overall in identifying good (unstripped) and bad (stripped) screw insertions, with their assessments of screw insertions being accurate 86% of the time compared to 62% for surgeons.

**Fig. 6:**
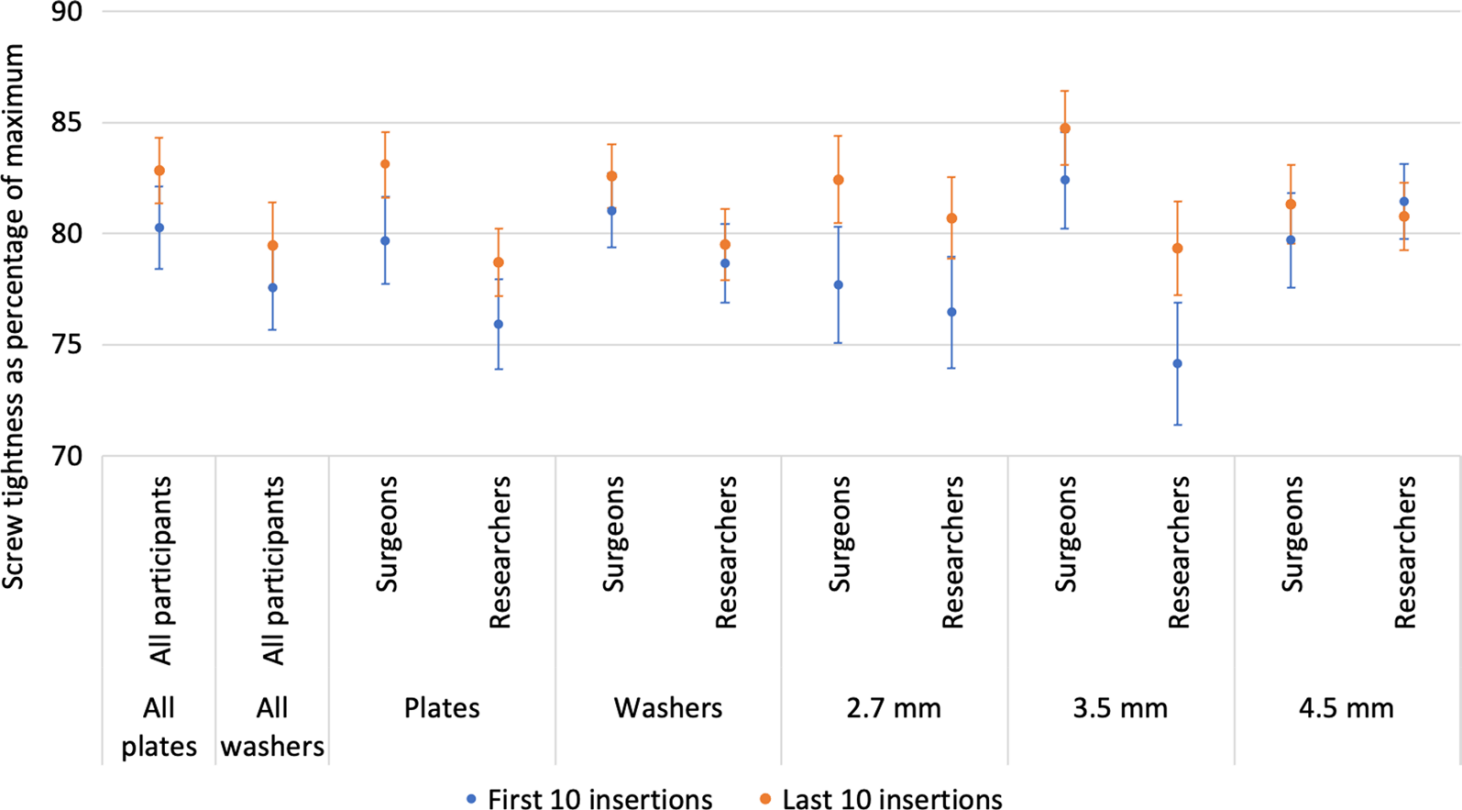
Learning effect—tightness achieved for the first 10 screws against the last 10 screws for surgeons and researchers for each variable

**Fig. 7:**
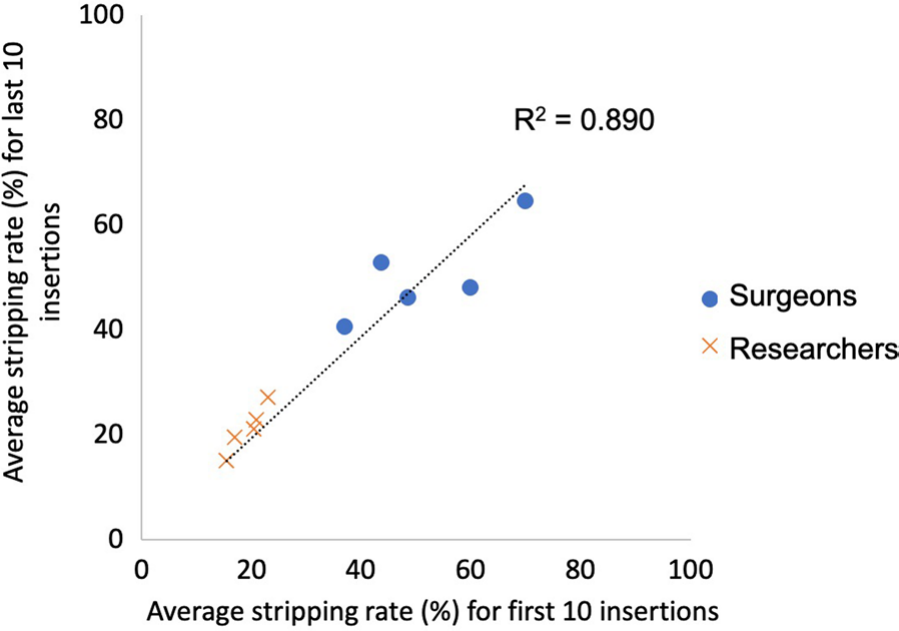
Learning effect—linear regression analysis of the mean average stripping rates for all 10 researchers and for all 10 surgeons, for the first 10 screws against the last 10 screws for each variable (five markers indicating 1. Plate fixation, 2. Washer fixation, 3. 2.5 mm screw diameter, 4. 3.5 mm screw diameter, and 5. 4.5 mm screw diameter): surgeons shown with blue circles and researchers with orange crosses

There was no significant change in screw tightness between the first 10 and last 10 screws inserted for any screw diameter or fixation technique (*p* = 0.058–0.821) (Fig. 6). A strong correlation between in the stripping rate for the first 10 insertions and the last 10 insertions was seen (*R*^2^ = 0.890) (Fig. 7). Using augmented screwdrivers led to a reduction in the stripping rate for surgeons (*p* = 0.162) and researchers (*p* = 0.001) (Table 2).

**Table 2:** Tightness and stripping rates before and with screwdriver augmentation for surgeons and researchers with the 1st, 5th and 10th highest stripping rates

	Number of attempted insertions	Number of unstripped insertions	Stripping rate (%)	Statistical difference in stripping rate	Unstripped screw tightness (%) (95% CI)	Statistical difference in tightness
Surgeons						
Pre-augmentation	90	49	46	*p* = 0.162	77 (73–81)	*p* = 0.036
Augmentation	90	56	38		83 (79–86)	
Researchers						
Pre-augmentation	90	70	22	*p* = 0.001	76 (71–81)	*p* = 0.472
Augmentation	90	86	4		74 (71–77)	

## Discussion

Within this study, surgeons showed a different ability from researchers in controlling screw insertion. There was a spectrum of abilities within both groups, with some surgeons and researchers generating very consistent screw tightness and minimal stripping rates, though both groups had participants who were insensitive to detecting stripping. Our findings raise concerns about the validity of methods in biomechanical research when insertion torque is neither recorded nor reported given the rates of poor screw insertion. Studies exclusively involving surgeons may generate more clinically transferable findings by mimicking clinical conditions more accurately; however the higher rate of stripped insertions that might occur during the experimentation could introduce into the methods an underappreciated confounder given the reduced compression generated and reduced pullout strength of stripped screws [9, 10] and their impact on fracture healing [19]. Additionally, our findings highlight the potential need for formalised screw insertion training in both the education of surgeons and biomechanical researchers.

This is the first study comparing tightness and stripping rates for different fixation methods and screw diameters. The same stripping rate was seen for both plate and washer fixation with average unstripped tightness close to the optimum tightness, defined as being between 70 and 80% of the stripping torque [9, 10]. Smaller diameter screws were tightened to a greater percentage of the stripping torque than larger screws, with a greater stripping rate, perhaps as the force required to exceed the stripping torque could be applied more effortlessly. Great awareness of the risks of poor screw insertion appears to be needed when inserting 2.7 mm screw given the high stripping rate seen. Experience did not impact on screw tightness nor stripping rates for either group, potentially highlighting how an individual develops their own technique that does not significantly change over time. This may occur due to a lack of attention on performance or an inability to critique it, alongside a general trivialisation within the surgical community of screw insertion—that it is easy and does not require special consideration or training. The lack of previous research into surgeon performance [1] and the absence of these techniques in surgical curricula [2], supports this argument. This study emphasises the need for improved awareness and training of basic biomechanical procedures, such as tightening a screw without stripping the screw hole, or at least recognising when that happens.

Good screw fixation is reliant on the ability to contemporaneously critique a screw’s insertion to ensure the screw will perform as intended. If insertion is felt to be poor, alternative remedies, though often suboptimal, can be enacted if the screw hole has been stripped. These corrections include, for example, re-siting a screw or inserting a larger diameter screw. Both groups in this study, on average, correctly showed a significant difference in the confidence of a screw’s holding ability between unstripped and stripped screw insertions. However, researchers were appropriately less confident when screw holes were stripped. Building on the need for sensitivity to inappropriate tightening, the capability to determine when a screw insertion was stripped differed between researchers and surgeons. Accuracy in detecting stripping highlighted that some participants were insensitive to stripping, a finding seen before by Stoesz et al., who found that more than 90% of stripped screw insertions went undetected by surgeons [15]. Additionally, some participants believed a screw to be poorly inserted when in fact it had not stripped the screw hole. Our findings show that proprioceptive assessment appears variable amongst surgeons and researchers, but also that more focus is likely to be needed on training both researchers and surgeons on how to insert screws correctly and what they should be feeling for during insertion.

There was weak evidence of increasing tightness with more insertions, with no change in the stripping rate between the comparative groups of the first third and last third of insertions. This echoes the findings of a larger study into screw insertion variables that showed for all but a few of the tested conditions there was no increase in tightness with more insertions, and that the performance when inserting the first 10 screws was representative of a larger number of screw insertions [3]. More screws may reflect an individual technique with more accuracy; however using 10 screws to test an insertion condition seems to be sufficient as the tightness does not generally change with more insertions, nor does the stripping rate. These findings can be used to reduce the volume of materials needed in future studies into screw insertion technique.

Awareness of the applied torque value and when optimum torque has been reached was seen to reduce stripping rates. Gustafson et al. investigated surgeons inserting screws into 0.1 g/cm^3^ artificial bone models finding a significant (*p* < 0.001) reduction in the stripping rate from 42 to 15% when they were unblinded to the applied torque [14]. Bone characteristics and screw geometries can be used to estimate the stripping torque for a screw hole prior to insertion, enabling prediction of an optimum torque that represents 70–80% of the stripping torque [9, 10]. Using these predictions and augmenting screwdrivers to indicate the torque as it is applied, shows promise for improving osteosynthesis.

One of the key strengths of this study is the number of screws inserted, and thus the power of this study, as this is considerably more than any previous work examining screw insertion outcomes [1]. The transferability of the findings of our study is greatly enhanced by having 20 individuals each insert 180 screws, and six participants inserting a further 60 screws each (total *n* = 3960). The similarity between the tightness of the first ten screws inserted and the last 10 screws for a test variable shows that future studies may appropriately investigate a situation with the insertion of only ten screws. However, even ten screws under the same conditions are more than the number used in most previous biomechanical studies into screw fixation [1]. The apparatus used enabled testing of screw diameters and augmentation in a repeatable fashion, which is especially important given the effects other factors can have. A previous study has shown significant and unpredictable differences in the tightness of screws and stripping rates depending on the conditions under which screws are inserted [3]. Thus, all variables, such as cortical thickness, use of gloves and bone density, were appropriately controlled during experimentation to not introduce confounders.

There were limitations with the study, including that the model used for testing mimicked low density bone, with only unicortical fixation performed which may not be representative of the majority of screw insertions in clinical practice. However, previous work has shown that screw techniques in human cadaveric models mimic those of artificial bone [3]. Unicortical insertion was used to reduce the amount of artificial bone needed, which will not model most clinical fixations, though bicortical screw fixation has been shown to perform comparably to unicortical fixation; it is the total cortical thickness that effects screw behaviour rather than whether the cortices are split [20]. Furthermore, the purpose of this study was not to assess a specific clinical scenario, but to have a standardised model to investigate the variations in techniques. Despite the bone density and the stripping torques being low, several participants were able to repeatably insert screws correctly, showing that good fixation for the conditions was possible, and that the poor results seen for some, unfortunately, cannot be explained by the testing arrangement. Detailed analysis of causative factors in individual performances was beyond the scope of this study, though could prove useful for future research to identify if there are modifiable risk factors for poor performance. Some aspects, such as maximum torque strength have been reviewed, but not shown in small samples to be related with changes in screw tightness [21]. Finally, no assessment was performed of the strength of the created constructs, though it has been established that with excessive torques, a construct is greatly weakened [9, 10, 19].

## Conclusions

The sample of surgeons and researchers analysed frequently showed different screw tightness under the same conditions, with significantly greater rates of screw hole stripping by surgeons. With the majority of screw research being performed by non-surgical, biomechanical researchers, there may be a failure to replicate in vitro findings if the skills of the surgeons differ greater from those making research discoveries. Greater attention to teaching optimal screw insertion to both surgeons and researchers is warranted alongside further investigation into the clinical use of augmented screwdrivers to indicate optimum tightness.

## Data Availability

All data are available in an online repository https://doi.org/10.15125/BATH-00955.
